# Comparative molecular analyses of *Borrelia burgdorferi* sensu stricto strains B31 and N40D10/E9 and determination of their pathogenicity

**DOI:** 10.1186/1471-2180-12-157

**Published:** 2012-07-30

**Authors:** Kamfai Chan, Mehwish Awan, Stephen W Barthold, Nikhat Parveen

**Affiliations:** 1 Department of Microbiology and Molecular Genetics, University of Medicine and Dentistry of New Jersey, New Jersey Medical School, 225 Warren Street, Newark, NJ 07103–3535, USA; 2 Center for Comparative Medicine, Schools of Medicine and Veterinary Medicine, University of California Davis, One Shields Avenue, Davis, CA, 95616

**Keywords:** *Borrelia burgdorferi* strains, B31, N40, Adherence, Pathogenesis, Tissue colonization, Lyme disease

## Abstract

**Background:**

Lyme disease in the United States is caused primarily by *B. burgdorferi* sensu stricto while other species are also prevalent in Europe. Genetic techniques have identified several chromosomal and plasmid-borne regulatory and virulence factors involved in Lyme pathogenesis. B31 and N40 are two widely studied strains of *B. burgdorferi,* which belong to two different 16 S-23 S rRNA spacer types (RST) and outer surface protein C (OspC) allelic groups. However, the presence of several known virulence factors in N40 has not been investigated. This is the first comprehensive study that compared these two strains both *in vitro* and using the mouse model of infection.

**Results:**

Phylogenetic analyses predict B31 to be more infectious. However, our studies here indicate that N40D10/E9 is more infectious than the B31 strain at lower doses of inoculation in the susceptible C3H mice. Based-upon a careful analyses of known adhesins of these strains, it is predicted that the absence of a known fibronectin-glycosaminoglycan binding adhesin, *bbk32*, in the N40 strain could at least partially be responsible for reduction in its binding to Vero cells *in vitro*. Nevertheless, this difference does not affect the infectivity of N40D10/E9 strain. The genes encoding known regulatory and virulence factors critical for pathogenesis were detected in both strains. Differences in the protein profiles of these *B. burgdorferi* strains *in vitro* suggest that the novel, differentially expressed molecules may affect infectivity of *B. burgdorferi*. Further exacerbation of these molecular differences *in vivo* could affect the pathogenesis of spirochete strains.

**Conclusion:**

Based upon the studies here, it can be predicted that N40D10/E9 disseminated infection at lower doses may be enhanced by its lower binding to epithelial cells at the site of inoculation due to the absence of BBK32. We suggest that complete molecular analyses of virulence factors followed by their evaluation using the mouse infection model should form the basis of determining infectivity and pathogenicity of different strains rather than simple phylogenetic group analyses. This study further emphasizes a need to investigate multiple invasive strains of *B. burgdorferi* to fully appreciate the pathogenic mechanisms that contribute to Lyme disease manifestations.

## Background

Lyme disease is a multisystemic disease caused by *Borrelia burgdorferi,* which is transmitted by *Ixodes* ticks in the United States of America [1,2]. The earliest clinical sign of Lyme disease is an expanding rash at the site of tick bite known as erythema migrans [3]. If left untreated, infection with Lyme spirochetes can disseminate to joints, heart, skin and central nervous system [3]. A resulting persistent infection of the host can then result in the development of arthritis, carditis, or neuroborreliosis [4]. Arthritis is the primary manifestation of late and chronic Lyme disease by *B. burgdorferi* sensu stricto, the predominant genospecies in the United States.

The genetic basis of bacterial virulence and disease has been investigated in a large number of Gram-negative and Gram-positive bacteria in the last three decades and major virulence factors of each microbe have been identified. These studies have shown that various strains of bacterial pathogens often exhibit different levels of pathogenicity and disease manifestations in the hosts. In most cases, the high pathogenicity is associated with specific variations in the set of virulence factors [5-11]. In many microbes, the respective virulence factor-encoding genes are clustered together in specific regions defined as pathogenicity islands [12]. Strains of *B. burgdorferi* show a high variation in their ability to cause disseminated infection. Since genetic studies have been developed in this spirochete only in the past decade, classification based upon its virulence factor diversity has not yet been fully developed. Furthermore, the presence of a segmented genome has hampered studies with different spirochete strains. However, *B. burgdorferi* sensu stricto strains have been divided into different groups either on the basis of allelic variation in the Outer surface protein C (OspC), which is essential for causing infection in the mammalian hosts [13-16], or the polymerase chain reaction (PCR) and restriction fragment length polymorphism analysis of 16 S-23 S rRNA spacer types (RST). Furthermore, *ospC* or RST groups were used as markers to determine pathogenicity of different *B. burgdorferi* strains with only some groups considered invasive [17-24].

Studies involving the two most widely investigated strains, B31 and N40, have contributed significantly to the understanding of Lyme disease pathogenesis and assessment of the virulence factors of *B. burgdorferi*[25-27]. B31 and N40 strains were isolated from *Ixodes scapularis* ticks from Shelter Island and Westchester county of New York, respectively, and both are highly infectious in the mouse model [2,28]. Indeed, N40 strain was selected for its high pathogenicity from a large number of isolates recovered from ticks by Durland Fish. By a thorough genetic analysis of various clones of N40 used in various laboratories, we have recently shown that the original culture was a mixed culture and different researchers isolated two different clones independently and retained the original name, N40, for both [29]. The clones designated as cN40 and the sequenced N40B are the derivatives of the same strain and N40 clone D10/E9 (N40D10/E9) and N40C appear to be derivatives of the second strain that is different from cN40/N40B. Comparative genomic analyses have indicated substantial genetic diversity between B31 and N40B [30]. For example, N40B possesses a smaller linear chromosome and contains fewer endogenous plasmids than the B31 strain [30]. To avoid further confusion, we will define specific N40 strains described above and in our recently published paper to determine their relevance to the published literature on these strains [29]. Genotyping by the pulsed field gel electrophoresis (PFGE) method defined the B31 strain as PFG type B and the cN40 strain as PFG type E [31]. In addition, the B31 strain belongs to the RST1 group while the cN40 strain is in the RST3 group [23]. Interestingly, a higher proportion of the *B. burgdorferi* strains isolated from patients with disseminated Lyme disease belong to the RST1 group [23,24,32]. Therefore, several researchers have concluded that RST1 group *B. burgdorferi* strains are more infectious and pathogenic than those of other groups [32,33]. Although several strains belonging to the RST3 group cause disseminated infection infrequently [23,24,32], a further subclassification showed that some strains of RST3B can result in a significant disease [32]. Based upon comparative analyses of the selected *B. burgdorferi ospC* sequence and RST1 and RST3 group strains [21,32-34] it is sometimes erroneously concluded that cN40 (RST3B, *ospC* type E) or N40D10/E9 (RST3B, *ospC* type M) could be less virulent than the B31 (RST1, *ospC* type A) strain. However, numerous experimental studies have established that cN40 is highly pathogenic in various animal models [35-39]. We, and others, have been studying N40D10/E9 for more than a decade and found that this strain is also highly virulent in the mouse model. However, a systematic comparative analysis of N40 strains with the sequenced B31 strain was not conducted to determine if both are equally pathogenic or N40 strains are indeed less virulent than B31.

Adherence is often the first step in establishment of infection by pathogenic bacteria and colonization of host tissues. Lyme spirochetes are primarily extracellular, tissue tropic pathogens and are found adherent to the host cells and extracellular matrix both in the patients’ samples and mouse tissue sections, suggesting important roles played by binding mechanisms in tissue colonization. Furthermore, binding to host cells is likely to be critical for *B. burgdorferi* facilitating selection of suitable niche for their growth and promoting colonization of the specific tissues. Binding to particular tissues could then allow Lyme spirochetes to escape immune system in some cases [40]. Indeed, a variety of host receptors and spirochetal adhesins are implicated in adherence and tissue colonization [41-46]**.** Glycosaminoglycans (GAGs) are the most abundant ubiquitously expressed molecules on mammalian cell surfaces and as components of the extracellular matrix (ECM). They are likely to be the first molecules recognized by *B. burgdorferi* on the host cell surface due to initial charge-based interactions. GAGs are long, unbranched polysaccharide molecules consisting of disaccharide repeats of modified sugars and uronic acids [47]. Based on the degree of sulfation and the composition of the disaccharides, they are classified into heparin, heparan sulfate, chondroitin sulfate A, dermatan sulfate, chondroitin sulfate C, and keratan sulfate [48]. GAGs are usually covalently linked to protein cores to form proteoglycans. A previous study has shown that Lyme spirochetes do not recognize keratan sulfate [49]. In *B. burgdorferi*, several adhesins recognize GAGs and proteoglycans. We previously identified *Borrelia* glycosaminoglycan-binding protein (Bgp), an outer membrane protein that binds heparin and dermatan sulfate, and facilitates binding of *B. burgdorferi* to epithelial cells and glial cells [50]. In addition, the *B. burgdorferi* surface lipoproteins decorin-binding proteins A and B (DbpA and DbpB) recognize both decorin and dermatan sulfate [43,51,52]. An additional adhesin, BBK32 (fibronectin binding protein) is a surface lipoprotein that can bind both fibronectin and GAGs to promote binding of *B. burgdorferi* to various mammalian cells [41,53]. P66 recognizes the integral membrane integrin receptor and was first identified as an adhesin in the N40D10/E9 strain [54,55] and was also shown to express in the B31 strain [56,57]. Hence, multiple adherence mechanisms are present in *B. burgdorferi* emphasizing its importance in causing multisystemic Lyme disease*.*

To evaluate the molecular mechanisms involved in *B. burgdorferi* tissue colonization and multisystemic disease during mammalian infection, many different types of host cell lines can be employed to investigate adherence [58-64]. For example, Vero cells, which were derived from monkey kidney epithelium [65], can be used as a representative of epithelial cells for studying GAGs-mediated adherence. The EA.hy926 cell line was derived from human umbilical vein endothelial cells, and it has been shown to express differentiated functions that are characteristics of human vascular endothelium [66,67]. C6 glioma cells were derived from rat central nervous system and were previously shown to display glycosaminoglycans, heparan sulfate and chondroitin sulfates, on their surface [43,61,68]. The T/C-28a2 cell line was developed from human chondrocyte cells [69], which were shown to express fibronectin, decorin and dermatan sulfate [70,71]. We have used these cell lines to compare the differential adherence abilities of N40D10/E9 and B31 strains.

The mouse is the natural host for *B. burgdorferi* and the laboratory mouse model has been used to study infectivity and pathogenicity of Lyme spirochetes. Different strains of immunocompetent mice develop different degrees of pathology upon infection with *B. burgdorferi*. For example, C57BL/6 mice develop mild carditis and arthritis even though colonization of the tissues is relatively similar to that of disease-susceptible C3H mice [72,73]. Arthritis development in BALB/c mice is dependent upon infectious dose, where higher doses caused more severe disease [72]. In contrast, C3H mice develop severe carditis and arthritis with low infectious doses [72,73]. Differential levels and types of localized cytokines production have been attributed to the disease severity in these strains of mice [74,75]. Although some laboratories use other mouse systems [76-80], C3H mice are ideal for discrimination of the infectivity and pathogenicity of different *B. burgdorferi* strains.

In this study, we assessed the presence of known critical virulence factor encoding genes in both B31 and N40D10/E9 strains. We employed various techniques for comparative analyses of B31 and N40D10/E9 strains to show that both spirochetes possess ability to bind to various mammalian cells *in vitro*, can colonize different tissues during infection and cause multisystemic disease in the immunocompetent C3H mice. Interestingly, N40D10/E9 is more infectious than B31 when lower dose of inoculum is used.

## Results

### *B. burgdorferi* strain B31 binds better to Vero epithelial cells than N40D10/E9

**Figure 1:**
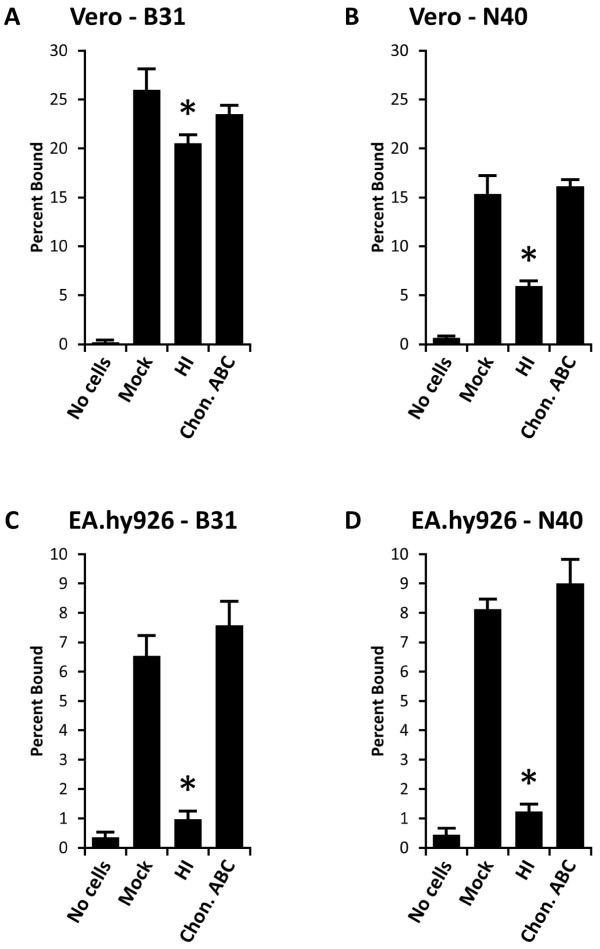
** Binding of *B. burgdorferi* strains B31 (A and C) and N40D10/E9 (B and D) to both Vero (epithelial) cells and EA.hy926 (endothelial) cells is mediated by heparan sulfate.** (**A**) and (**B**). Vero cell monolayers were pretreated with the buffer alone (Mock), or with the GAG lyases, heparinase I (HI) to remove heparan sulfate or chondroitinase ABC (Chon. ABC), to cleave chondroitin sulfates from the cell surfaces. Binding of B31 (A) to the Vero cells was significantly higher than that of the N40D10/E9 (B) strain. Although inhibition of binding of both N40D10/E9 and B31 was significant, reduction in binding was more pronounced by N40D10/E9 than B31 when Vero cells were treated with HI (p < 0.05). (**C**) and (**D**). EA.hy926 cell monolayers were mock-treated, or pretreated with HI or Chon. ABC enzymes. Removal of heparan sulfate from EA.hy926 cells eliminated the binding of both B31 and N40D10/E9 strains to these cells. The experiments were repeated at least three times using four replicates for each treatment. Each value represents the mean ± SD of quadruplicate samples. Asterisks indicate significant reduction (p < 0.05) in binding percentage compared to mock-treated cells as determined by t-test for pairwise comparison of samples with unequal variance.

It has been shown previously that *B. burgdorferi* strain N40D10/E9 binds efficiently to Vero epithelial cells [49,58]. A comparison of binding of the *B. burgdorferi* strains B31 and N40D10/E9 to Vero cell monolayers *in vitro* showed that 25% of B31 and 15% of N40D10/E9 spirochetes remained bound when the cells were mock-treated (Figures 1A and 1B). We previously showed that heparin-related molecules mediate binding of N40D10/E9 strains to the Vero cells [61,62]. When the cells were treated with heparinase I to cleave heparan sulfate from the cell surface and removed by washing, the binding of B31 was reduced by 20%. Although this binding reduction was statistically significant (p = 0.014) as determined by t-test, decrease in binding of N40D10/E9 to Vero cells was more pronounced with approximately 67% reduction when heparan sulfate was removed from cells by heparinase I (Figures 1A and 1B). Chondroitinase ABC can cleave chondroitin sulfate A, chondroitin sulfate B (dermatan sulfate), and chondroitin sulfate C [81]. However, there was no significant change in the binding of either B31 or N40D10/E9 strains when the Vero cells were treated with chondroitinase ABC, indicating that dermatan sulfate and other chondroitin sulfates do not contribute to the binding of Lyme spirochetes to these cells. Since *B. burgdorferi* does not bind keratan sulfate glycosaminoglycan [49], the remaining 80% residual binding of B31 and approximately 33% residual N40D10/E9 binding to Vero cells after heparan sulfate removal indicate that both strains may also bind to the Vero cells using a GAG-independent pathway. The role of these mechanism(s) is significantly higher in adherence of B31 to Vero cells. 

### Attachment of *B. burgdorferi* strains B31 and N40D10/E9 to EA.hy926 endothelial cells is also mediated by heparan sulfate

To study whether *B. burgdorferi* strains B31 and N40D10/E9 exhibit a similar pattern of interaction with endothelium, these spirochete strains were allowed to bind to EA.hy926 endothelial cells *in vitro*. Both strains showed lower and relatively similar levels of binding to EA.hy926 cells and 6.5% of B31 and 8% of N40D10/E9 remained bound to mock-treated EA.hy926 cells (Figures 1C and 1D). Treatment of EA.hy926 cells with heparinase I significantly and almost completely eliminated binding of both strains to these endothelial cells with a remnant adherence level (1% only) equivalent to that in the empty wells control (“no cells” in Figures 1C and 1D). Treatment with chondroitinase ABC did not affect binding of the spirochetes to the EA.hy926 cells relative to mock-treated endothelial cells, indicating that either EA.hy926 cells do not express chondroitin sulfates or these spirochete strains do not recognize chondroitin sulfates on EA.hy926 cells (Figures 1C and 1D). These results agree with our previous finding that heparan sulfate is the major receptor recognized by different Lyme spirochetes on EA.hy926 endothelial cells [61].

### Dermatan sulfate plays an important role in the binding of *B. burgdorferi* to C6 glioma and T/C-28a2 cells

**Figure 2:**
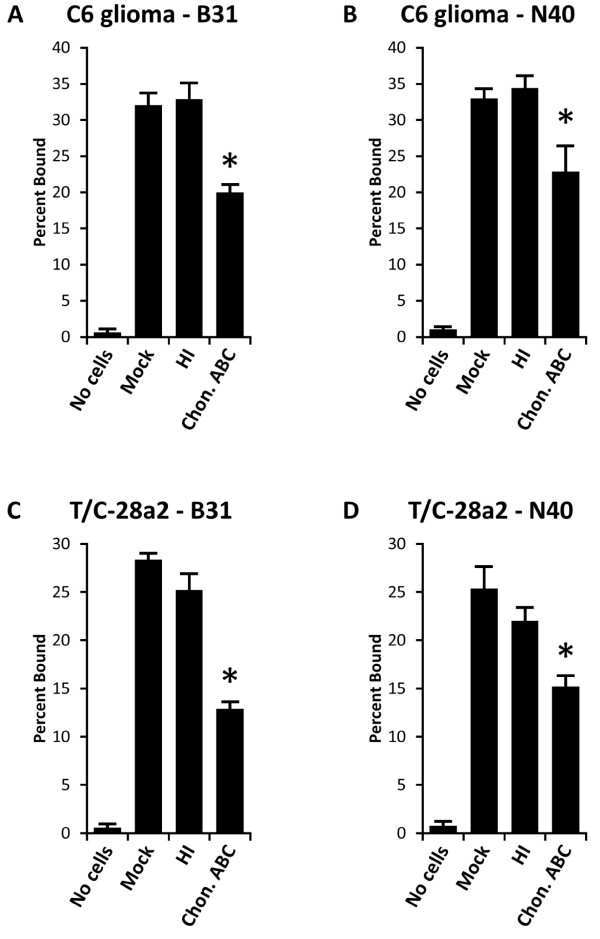
** Binding of *B. burgdorferi* strains B31 and N40D10/E9 to C6 glioma and T/C-28a2 chondrocyte cell monolayers was significantly reduced on pretreating these cells with chondroitinase ABC but remain unaffected on their pretreatment with heparinase I.** The experiments were repeated at least three times using four replicates for each treatment. Each value represents the mean ± SD of quadruplicate samples. Asterisks indicate significant reduction (p < 0.05) in binding percentage relative to mock-treated cells as determined by t-test for pairwise comparison of samples with unequal variance.

When *B. burgdorferi* strains B31 and N40D10/E9 were allowed to bind to mock-treated C6 glioma cells, approximately 32% of each strain of spirochetes bound to the C6 cells (Figures 2A and 2B). On treatment of C6 glioma cells with heparinase I, binding of both strains remained unaffected as compared to mock-treated cells (Figures 2A and 2B). Both strains showed significant reduction in binding to C6 glioma cells after chondroitin sulfate A, chondroitin sulfate B (dermatan sulfate), and chondroitin sulfate C were removed by pretreatment of these cells with chondroitinase ABC (Figures 2A and 2B). However, we have previously shown that several *B. burgdorferi* strains, including N40D10/E9, barely recognize chondroitin sulfate A and chondroitin sulfate C [49,61,62]. Therefore, we conclude that the adherence of both *B. burgdorferi* strains to glial cells was mediated primarily by dermatan sulfate. 

Similarly, binding of B31 to T/C-28a2 chondrocyte cells was reduced, by the treatment of chondroitinase ABC, from 28% to 13% (Figure 2C). N40D10/E9 binding was reduced from 26% to 15% (Figure 2D). Since heparinase I had no significant effect on the binding of both strains to T/C-28a2 cells (Figures 2C and 2D), adherence of B31 and N40D10/E9 to chondrocyte cells appeared to be mediated primarily by dermatan sulfate and receptor(s) other than GAGs.

### Majority of the known virulence factors encoding genes of the B31 strain are also present in the N40D10/E9 strain

**Figure 3:**
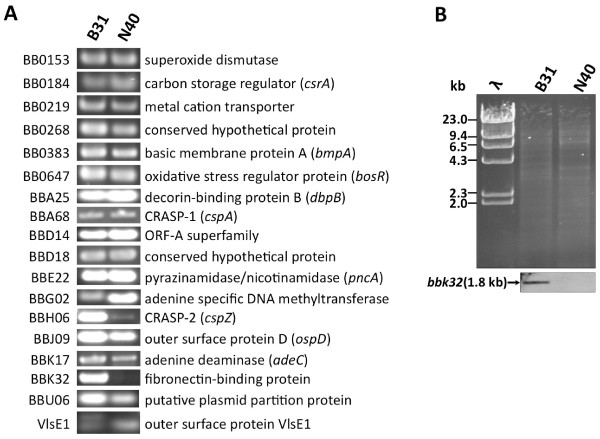
** The gene homologous to the *bbk32* was not detected in N40D10/E9 strain by PCR and Southern hybridization.** (**A**). Using the primers in Additional Table 1, complete genes *bb0153*, *bb0184* (*csrA*), *bb0219, bb0268, bb0383* (*bmpA*), *bb0647* (*bosR*), *bba25* (*dbpB*), *bba68* (*cspA*), *bbd14, bbd18, bbe22* (*pncA*), *bbg02, bbh06* (*cspZ*), *bbj09* (*ospD*), *bbk17* (*adeC*), *bbu06*, and partial *vlsE1* gene (using internal conserved primers) were amplified by PCR using B31 and N40D10/E9 strains genomic DNA as template. The *bbk32* gene was amplified from B31 genomic DNA, however, PCR product was not detected in the N40D10/E9 strain. (**B**) Southern blot of *Eco*R1-digested genomic DNA of both strains (top) was hybridized with the probe prepared using the *bbk32* PCR product from B31. An approximately 1.8 kb size fragment was detected only in B31, as expected, but not in the N40D10/E9 genomic DNA containing lane.

Since the first demonstration of the essential role of OspC in mammalian infection using the genetic approach in 2004 [13], several molecules have been shown to be important for causing infection and disease in the mouse model [44,82-100]. The N40D10/E9 strain is not yet sequenced and its plasmid profile is different from the B31 strain [29]. Therefore, limited genomic and proteomic analyses were conducted to compare these two strains. To determine if these two *B. burgdorferi* strains show differences in the presence of genes encoding known adhesins, other virulence factors and their regulatory proteins, we amplified these genes by PCR to investigate and differentiate these two strains. Interestingly, all previously established virulence factors encoding genes were present both in B31 [101] and N40D10/E9 strains except the *bbk32* gene (Figure 3A). Two different size PCR products were observed in B31 when internal VlsE1 primers were used for gene amplification. This agrees with the presence of two homologs shown in the genome website, *bbf0041* and *bbj51* but only *bbf0041* (VlsE1) is functional since *bbj51* has a stop codon after 57 amino acids. However, only one *vlsE1* gene was detected in N40D10/E9 probably because lp38, which contains *bbj51,* is missing in this strain [29]. 

In another study, we compared two important, highly variable virulence factors of *B. burgdorferi*, OspC and DbpA. As expected, both of these molecules are present in both spirochete strains but showed high sequence variation [29]. Therefore, irrespective of the phylogenetic grouping of these strains using RST and OspC categorization, the presence of known virulence factors in both strains suggests that B31 and N40D10/E9 could possibly exhibit similar levels of pathogenicity. Furthermore, although BBK32 is an adhesin [41], previous studies showed that its absence results in a subtle infectivity defect, exhibiting disease attenuation only at low dose of infection [45,102,103].

### Divergence of fibronectin-binding adhesin gene *bbk32* in N40D10/E9 strain

BBK32 could possibly contribute to the adherence-mediated tissue colonization in B31 as compared to N40D10/E9 strain but a negative PCR result is not sufficient to demonstrate this difference. Since sequence divergence at the priming sites may lead to unsuccessful PCR amplification, Southern hybridization was conducted to determine the presence of a homolog of *bbk32* gene in the N40D10/E9 strain. Absence of a band in N40 even under low stringency conditions (data not shown) indicated that either *bbk32* homolog in the N40D10/E9 strain was absent or had substantial DNA sequence divergence from that in the B31 strain (Figure 3B). Therefore, irrespective of the presence of BBK32, the two *B. burgdorferi* strains examined here (B31 and N40D10/E9) show similar levels of binding to most cells, indicating redundancy of function. However, BBK32 may contribute to the binding of Lyme spirochetes to specific cell line(s), such as Vero cells, and potentially to epithelial cells *in vivo*.

### B31 and N40D10/E9 showed remarkably different protein expression profiles

**Figure 4:**
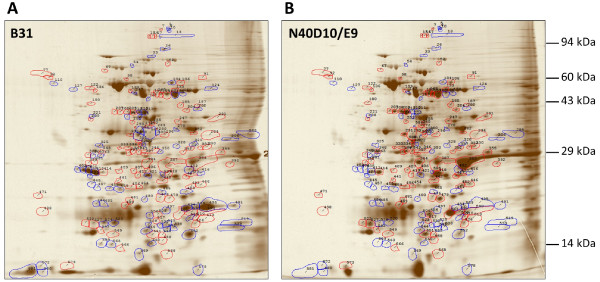
** Two-dimensional gel electrophoresis of B31 and N40D10/E9 strains total proteins.** Polypeptide spots with increased relative abundance (more than 1.7 fold increase) in B31 versus N40D10/E9 are outlined in blue while spots with decreased relative abundance (more than 1.7 fold decrease) in B31 versus N40 are outlined in red. Several of these spots were sent for MALDI-MS analysis.

Although known virulence factors are present in both B31 and N40D10/E9 strains (Figure 3A), they only represent the molecular profile of previously identified virulence factors and molecules associated with infectivity. Therefore, it would be erroneous to conclude that they represent the full repertoire of the virulence factors of *B. burgdorferi* that play important roles during pathogenesis in the mammalian host. Since the N40D10/E9 genome is not sequenced, it is possible that this strain contains additional virulence factors that play a role in Lyme pathogenesis. Alternatively, specific virulence factors may be expressed differentially in the two strains. To discern the differences in the protein profiles of these two strains, a comparative analysis of proteins expressed *in vitro* was conducted by a two-dimensional protein gel electrophoresis and is shown in Figures 4A and 4B. Intensity of individual polypeptide spots was measured after gel electrophoresis. For each polypeptide, the relative abundance was calculated from individual spot intensity against that of all measured polypeptide spots. The polypeptides that were expressed at significantly differential levels in the two strains are summarized in Table 1. Out of 591 polypeptide spots analyzed, 26 were found to have at least a 10-fold increase in relative abundance in B31 than in N40D10/E9. On the other hand, 22 polypeptide spots had at least a 10-fold increase in relative abundance in N40D10/E9 than in B31. The increase in relative abundance indicated that the polypeptides could be uniquely expressed in a particular strain, or they could be severely repressed in the other strain. One or more of the proteins expressed uniquely in N40D10/E9 or at higher levels in this strain during infection could contribute to the higher level of infectivity and disease severity relative to dose of infection of the N40D10/E9 strain.

**Table 1:** Polypeptide spots that showed at least a 10-fold increase in relative abundance in B31 or N40D10/E9 on 2D protein gel

**Spot #**	**pI**	**MW (kDa)**	**Relative abundance in B31, and N40 (%)**	**Fold change B31 vs N40**	**Identification MALDI-MS analyses (SwissProt or NCBI accession #)**	**Spot #**	**pI**	**MW (kDa)**	**Relative abundance in B31, and N40 (%)**	**Fold change N40 vs B31**	**Identification MALDI-MS analyses (Swi**ss**Prot or NCBI accession #)**
**33**	6.2	88.96	0.036, 0.003	**11.2**		**136**	5.6	64.58	0.002, 0.029	**14.7**	
**110**	5.1	63.92	0.050, 0.003	**15.1**		**208**	5.8	53.07	0.015, 0.340	**22.7**	
**127**	5.3	65.24	0.037, 0.003	**11.5**		**231**	6.9	52.81	0.019, 0.226	**11.8**	
**211**	6.1	55,65	0.875, 0.048	**18.0**		**272**	6.2	46.29	0.000, 0.054	**685.4**	*Flagellin (GI:120230), Basic membrane protein A (GI:3913169)
**225**	6.1	57.07	0.193, 0.005	**35.3**		**293**	6.0	43.53	0.000, 0.170	**698.2**	*Flagellin (GI:120230)
**325**	5.6	38.32	0.114, 0.010	**11.3**		**311**	6.0	39.99	0.005, 0.165	**30.6**	
**403**	5.4	31.03	0.071, 0.002	**29.1**		**347**	6.0	35.06	0.003, 0.185	**59.8**	
**404**	5.4	31.00	0.404, 0.003	**124.1**	OspD (GI:495462)	**348**	5.6	34.95	0.007, 0.258	**36.3**	
**405**	5.5	28.78	1.006, 0.031	**32.7**		**349**	6.0	34.36	0.003, 0.095	**32.4**	
**458**	5.7	26.07	0.051, 0.003	**15.2**		**352**	6.5	34.25	0.003, 0.034	**12.0**	
**463**	6.5	25.58	0.107, 0.007	**15.4**		**354**	5.9	34.62	0.004, 0.207	**47.9**	
**465**	6.3	25.49	0.077, 0.006	**13.2**		**381**	5.6	29.61	0.003, 0.032	**10.4**	
**491**	7.0	22.69	0.356, 0.012	**29.8**		**406**	6.2	28.54	0.006, 0.081	**14.0**	
**494**	6.5	23.16	1.400, 0.062	**22.8**		**418**	6.3	27.97	0.089, 1.927	**21.6**	
**495**	6.7	23.13	6.875, 0.025	**278.1**	Outer surface protein C (GI:3914248)	**452**	6.6	26.33	0.006, 0.246	**42.5**	30S ribosomal protein S4 (B7J2H5) Phosphoglycolate phosphatase (GI:226320487), and hypothetical (GI:226315606)
**497**	6.3	22.87	0.262, 0.022	**12.1**		**479**	6.4	24.51	0.060, 0.648	**10.7**	
**519**	7.1	20.08	0.734, 0.027	**26.8**		**501**	6.5	22.47	0.030, 1.956	**64.6**	Same as 505
**525**	6.4	21.03	0.234, 0.008	**30.9**	Neutrophil activating protein (GI:15595035)	**505**	6.3	22.33	0.017, 0.570	**34.0**	OspC (GI: 226246807) Neutrophil activating protein (GI:15595035)
**528**	6.2	20.95	0.068, 0.004	**15.9**		**517**	6.2	21.41	0.002, 0.095	**54.4**	
**541**	6.4	20.31	0.097, 0.005	**20.8**		**543**	5.6	19.67	0.006, 0.072	**11.9**	
**559**	5.6	17.70	0.137, 0.008	**17.2**		**551**	6.2	19.51	0.075, 0.762	**10.2**	
**581**	4.9	11.91	2.069, 0.048	**42.7**	6.6 kDa lipoprotein (GI:1477781)	**573**	5.3	14.07	0.005, 0.255	**55.0**	
**585**	6.3	28.02	0.125, 0.010	**12.2**							
**586**	6.1	44.19	0.357, 0.001	**674.8**	*Flagellin (GI:120230)						
**587**	6.1	44.41	0.209, 0.000	**765.7**							
**588**	6.1	41.54	0.276, 0.001	**527.4**							

* Flagellin appears to be produced at equivalent levels in both strains but fold change depicted is higher for respective spots due to slight mobility differences of this protein in B31 and N40D10/E9 gels.

To further evaluate the differences in the proteins that are differentially expressed in the two strains, a limited MALDI mass spectrometric (MALDI-MS) analysis of selected protein spots was conducted. The proteins identified by MALDI-MS are listed in Table 1. Interestingly, three protein spots of slightly different mobility, number 586 in B31 and numbers 272 and 293 in N40D10/E9, were found to be more abundant (>650 times) than that of the equivalent spots in the compared strain. Our MALDI-MS analysis (Table 1) identified them as flagellin proteins. We amplified the flagellin gene (*bb0147*) from B31 and N40D10/E9 strains and sequenced the PCR product from the N40 strain. Sequence analysis showed a single amino acid change resulting in slight difference in the pI of the two proteins. This could affect mobility of the flagellin of each strain slightly on a 2D gel with each appearing as more abundant protein relative to the other *B. burgdorferi* strain (Figure 4 and Table 1).

### N40D10/E9 is more infectious than B31 in immunocompetent C3H mice

To determine if the B31 strain is more infectious and pathogenic than our N40D10/E9 strain, we used the susceptible C3H mouse infection model. By using different doses of *B. burgdorferi* strains injected subcutaneously into immunocompetent C3H mice, we determined the relative infectivity of each strain. Two weeks after inoculation, mice were euthanized, and diameters of tibiotarsal joints measured. Cultures examined microscopically from the blood, skin at the injection site, ear, joint, heart, and bladder were found to be positive from fewer tissues for the B31 strain than N40D10/E9 at a lower inoculum (Table 2) and based upon these results, the calculated median infectious dose (ID_50_) for B31 and N40D10/E9 were 371 and 46, respectively.

**Table 2:** Colonization of C3H mice tissues by B31 or N40D10/E9 strains examined two weeks after inoculation

**Strain**	**Inoculum**	**Recovery of*B. burgdorferi*from mouse tissues**							**ID**_**50**_
		**Blood**	**Injection site**	**Ear**	**Left joint**	**Heart**	**Bladder**	**Total**	
**B31**	10	0/3	0/3	0/3	0/3	0/3	0/3	0/18	
	10^2^	0/3	0/3	0/3	2/3	0/3	0/3	2/18	371
	10^3^	2/2	1/2	2/2	2/2	1/2	2/2	10/12	
	10^4^	2/2	2/2	2/2	2/2	1/2	2/2	11/12	
**N40**	10	1/3	2/3	2/3	0/3*	1/3	2/3	8/18	
	10^2^	3/3	2/3	2/3	0/3*	2/3	2/3	11/18	46
	10^3^	2/2	2/2	2/2	0/2*	1/2	2/2	9/12	
	10^4^	2/2	2/2	2/2	1/2	1/2	2/2	10/12	

Asterisks indicate that the cultures were contaminated.

**Figure 5:**
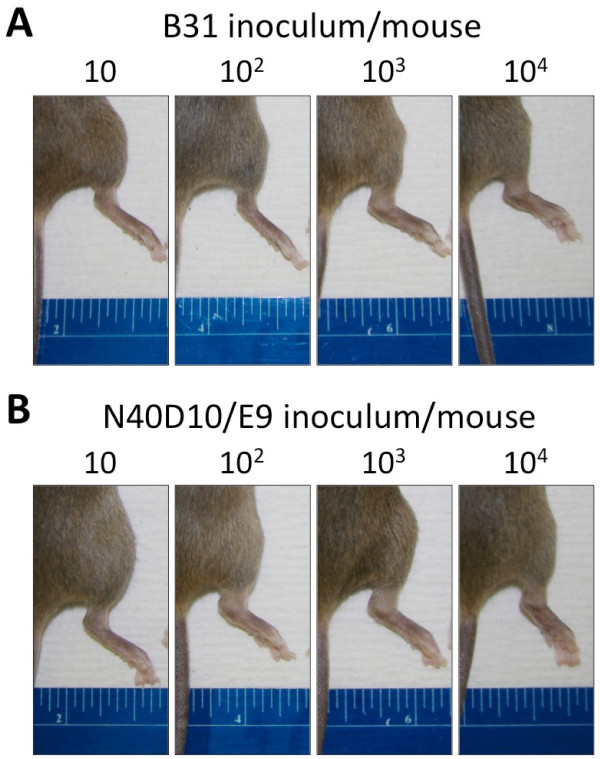
** Tibiotarsal joint inflammation in C3H mice inoculated with the N40D10/E9 and B31 strains.** C3H mice were inoculated with a different inoculum dose (10, 10^2^, 10^3^, 10^4^ spirochetes) of B31 or N40D10/E9 strains. (**A**) Two weeks after injection, severe tibiotarsal joint swelling was evident only in mice infected with 10^3^ or 10^4^ of B31. (**B**) However, severe tibiotarsal joint swelling could be observed in mice infected with 10, 10^2^, 10^3^ or 10^4^ of N40D10/E9.

In addition to differences in the infectivity of these two strains, mice injected with B31 appeared to manifest less severe joint disease than those infected with N40D10/E9, as evidenced by severe joint swelling exhibited by this strain at lower doses of infection (Table 3 and Figure 5). This was further confirmed by histopathological examination of the joints of the infected mice, which indicated that N40D10/E9-infected mice developed severe joint disease at the lowest infectious dose (10^1^), whereas B31-infected mice primarily developed arthritis at 10^3^ and higher dose of infection of *B. burgdorferi* per mouse. Mice with joint disease had involvement of the knees as well as of the tibiotarsal joints. Tibiotarsal arthritis was characterized by the presence of numerous infiltrating neutrophilic leukocytes in the periarticular tissue, tendons, ligaments, and synovial lining, which was thickened due to proliferation of synovial cells. Synovial lumina contained variable numbers of exuded neutrophils (data not shown).

**Table 3:** Tibiotarsal joint swelling and histological examination of joint tissues

**Strain**	**Inoculum**	**Right joint diameter (mm) (Avg±SD)**	**Right Tibiotarsus inflammation**	**Right knee/Tibial crest (Tc) inflammation**
**B31**	10	4.07±0.06	-, ±, +	-, -, -
	10^2^	3.90±0.20	-, ±, +	-, -, + (Tc)
	10^3^	5.10±0.00	3+, 3+	+, +,
	10^4^	4.90±0.00	3+, 3+	+, +
**N40**	10	4.03±0.15	-, 2+, 3+	-, +, +
	10^2^	4.60±0.17	+, 2 to 3+, 3+	+(Tc), +, +
	10^3^	4.75±0.07	3+, 3+	+, +,
	10^4^	5.00±0.00	3+, 3+	+, +

Joint swelling of each dose was quantitated by average diameter of the right tibiotarsal joint of multiple mice with each dose group. Joint inflammation was scored from “-” (no arthritis) to “+++ (3+)” (severe arthritis) in the tibiotarsus for infected mice at each inoculation dose. Knee or Tibial crest (Tc) inflammation was recorded as “-” (no arthritis) to “+” (arthritis) of each infected mouse.

## Discussion

Study of infectious bacterial species involving more than one virulent strain provides a more complete picture of the pathogenesis of the organism. B31 and N40 are two of the most widely examined *B. burgdorferi* strains in the USA to study Lyme disease pathogenesis. In 1997, B31 was the first *B. burgdorferi* genome that was published [101]. We have recently determined that different laboratories use two different N40 strains under the same strain name [29]. The genome of N40B was completed recently [30] but is not fully published. Our N40D10/E9 clone derivative is not yet sequenced but our critical evaluation has indicated that these two N40 strains are quite different even though both of them were isolated from the same tick [29]. Indeed, based upon RST and *ospC* types, both N40 strains are predicted to be a much less pathogenic strain than B31 [23,32,33,98-100]. However, at least in one study, a higher percentage of mice infected with the sequenced N40 as compared to those with B31 strain developed myocarditis (100% versus 92%). In addition, N40 showed both higher level of colonization in joints and arthritic lesions than that by B31 strain (60% versus 13%) in the infected mice [104]. Such a comparative study has not been carried out with our N40D10/E9 strain. Therefore, we conducted thorough comparative analyses both *in vitro* and *in vivo* to assess their infectivity and ability to colonize various tissues and cause disease.

*B. burgdorferi* strains have been shown previously to bind to various mammalian cell types *in vitro* and *in vivo*[58,60-64]. In this study, we selected Vero, EA.hy926, C6 glioma, and T/C-28a2 as representatives of epithelial, vascular endothelial, glial, and chondrocyte cells to study adherence of spirochetes *in vitro*. With the exception of Vero epithelial cells, *B. burgdorferi* strain B31 and strain N40D10/E9 showed approximately the same level of *in vitro* binding to various mammalian cells in this study. These results indicate the two most studied *B. burgdorferi* strains, B31 and N40D10/E9, exhibit some differences in adherence despite sharing similar capability and mechanisms for adherence to various mammalian cells *in vitro*.

Binding of B31 is significantly higher on Vero cells than N40D10/E9, but heparinase I treatment of these cells reduced binding of N40 strain much more dramatically (Figures 1A and 1B). These results suggest that a higher expression of surface proteins in B31 than N40D10/E9 that show affinity for host receptors other than heparan sulfate may be facilitating the attachment of this strain to Vero cells. Indeed, our study identifies BBK32 as one such candidate. N40B and cN40 contain both lp36 and BBK32 while our N40D10/E9 strain lacks lp36. It is possible that the BBK32 homolog in N40D10/E9 was significantly different from the BBK32 of B31 both at DNA and protein level, and hence, may not carry out the same functions. This can also explain a higher level of binding of B31 strain to Vero cells and potentially other cell lines that are not part of this study since in addition to its ability to recognize GAGs, BBK32 is also a fibronectin-binding protein [41,53]. Interestingly, the N40 strain with published sequence is different from our N40D10/E9 clone. The sequenced N40 contains a *bbk32* gene, which is similar to the *bbk32* of B31 with 96% identity and 97% similarity with the B31 protein. In another study, we have shown that lp36, which contains the *bbk32* gene in the B31 strain, is missing only in our N40 strain [29]. It is likely that the BBK32 protein, and potentially other unidentified adhesins, may contribute to the binding of the B31 strain, and not of N40D10/E9. BBK32 may recognize fibronectin as a component of the extracellular matrix of the Vero cells.

A predicted higher pathogenicity of the B31 strain relative to the N40D10/E9 strain based-upon RST and *ospC* grouping contradicts both our results and the findings of other researchers, who have used N40 strains [23,35,105,106]. Thus, RST and one virulence factor (*ospC*) sequence comparison may be important for phylogenetic analysis but may not be suitable for drawing conclusions about the pathogenicity of a particular strain of *B. burgdorferi* without assessment of the virulence factors or actually conducting the experiments. However, due to development of genetic manipulation techniques for *B. burgdorferi* only in the last decade, the roles of only a few virulence factors have been determined, and a comprehensive analysis of multi-virulence loci of B31 and N40D10/E9 strains is not yet possible. Furthermore, a full repertoire of the virulence factors for Lyme spirochetes is still not determined even on the basis of the sequence homology with genes of other pathogens since spirochetes contain most unique genes [101,107]. Finally, genetic manipulation and evaluation of mutant *B. burgdorferi* strains remains very time consuming and difficult. Therefore, the pathogenicity of B31 and N40D10/E9 cannot be determined simply by multi-virulence locus sequence typing (MVLST) at present similar to that used for other pathogenic bacteria [5,6,108]. Therefore, we used an alternative approach to investigate the functions relevant to tissue colonization of B31 and N40D10/E9 strains *in vitro* and examined their virulence in the mouse model. Interestingly, even though it was possible to determine the molecular basis of adherence using the mammalian cell lines, we did not see a direct correlation of the ability of these strains to adhere to the mammalian cells *in vitro* and infectivity or pathogenicity in the mouse host. However, it is possible that a lower adherence of the N40D10/E9 strain to epithelial cells at the site of inoculation enhances its dissemination to different tissues. These findings indicated that both *in vitro* and *in vivo* complementary approaches should be used to study different aspects of host-bacterial interactions and relevant determinations made without making generalized conclusions or extrapolations.

For further molecular differentiation of these two strains that may provide a possible hint about the differences we saw in their infectivity, we used PCR to determine the presence of genes encoding known virulence factors and associated proteins identified using a genetic approach in the last decade. We also evaluated the protein profiles of B31 and N40D10/E9 strains grown *in vitro*. Comparison of these two gels erroneously identified flagellin gene as different protein spots. This was depicted in the Table 1 as >650-fold change in the level of protein relative to the other strain. MALDI-MS analysis of the protein spots and sequence analysis of the N40D10/E9 flagellin gene were able to resolve this issue. The mobility shift of the flagellin in two gels is likely due to a single amino acid change resulting in slight difference in the pI of protein in B31 and N40D10/E9 strains. In addition to BBK32, comparative 2D-protein gel electrophoresis analysis revealed a large number of proteins that were uniquely expressed in either the B31 or N40D10/E9 strain. Several of these proteins have been identified. For example, the outer surface protein D (OspD, polypeptide spot 404 in Table 1) is highly expressed in B31 but not in N40. OspD has been shown to be responsible for colonization of *B. burgdorferi* in the tick gut [109,110]. However, OspD is not essential for transmission of the spirochete from tick to mouse or during the infection of the mouse [109,110]. In the N40D10/E9 strain, expression of the outer surface protein C (OspC and/or neutrophil activating protein spots 501 and 505 in Table 1) is expressed at much higher levels compared to that in the B31 strain. OspC lipoprotein is required for successful early stages of mouse infection [111], and one study suggests that OspC can facilitate dissemination of *B. burgdorferi* during mouse infection [76]. Investigation of the expression of the proteins of the N40D10/E9 strain, which are expressed at higher levels *in vitro,* also in the host-adapted spirochetes may shed light on the virulence factors that contribute to the higher infectivity of the N40D10/E9 strain during mouse infection. These will form the foundation of future studies to identify other important virulence factors of *B. burgdorferi* using extensive molecular and genetic approaches.

## Conclusion

We conclude that N40D10/E9 is more infectious in C3H mouse model than B31 when a lower dose of inoculation is used for needle injection while both strains are highly pathogenic in this model system. Our studies also indicate that phylogenetic analysis is not sufficient but a comprehensive appraisal of virulence and critical regulatory factors as well as their evaluation *in vivo* should be used to determine pathogenesis of a particular Lyme spirochete strain. Furthermore, it is suggested that multiple strains should be used to fully understand the infection and pathogenic mechanisms involved in Lyme disease manifestations since some invasive strains may possess or express specific virulence factors differentially.

## Methods

### Bacterial strains and cell lines

B315A4 clones were obtained from the laboratory of Steven Norris at University of Texas, Houston. The N40D10/E9 strain was originally cloned and provided by John Leong at Tufts University Medical School, Boston. Low passage (less than six) *B. burgdorferi* strains B31 and N40 (from original clone D10/E9) were grown in Barbour-Stoenner-Kelly-II (BSK-II) medium [112] supplemented with 6% rabbit serum at 33°C.

Various mammalian cell lines for this study were cultured according to recommended conditions originally provided by the suppliers. Vero (monkey kidney epithelial) cells were cultured in RPMI 1640 supplemented with 10% NuSerum IV (BD Biosciences, Franklin Lakes, NJ). EA.hy926 (human endothelial) cells were cultured in DMEM supplemented with 10% fetal bovine serum (FBS) and 1% HAT nutrient supplement (Invitrogen, Carlsbad, CA). C6 (rat) glial cells were cultured in RPMI 1640 supplemented with 8% FBS. T/C-28a2 (human chondrocyte) cells [69] were cultured in a 1:1 mix of DMEM and Ham’s 12 medium supplemented with 10% FBS. All mammalian cells were grown at 37°C in 5% CO_2_ atmosphere.

### Radioactive labeling of *B. burgdorferi*

*B. burgdorferi* strains were labeled with ^35^ S isotope as previously described [38]. Briefly, *B. burgdorferi* was cultured in BSK-II medium supplemented with 6% rabbit serum and 100 μCi/ml ^35^ S] -cysteine and -methionine protein labeling mix (Perkin-Elmer, Waltham, MA) at 33°C until the density was between 5 × 10^7^ and 1 × 10^8^ spirochetes per ml. The bacteria were harvested by centrifugation at 5000 × g for 20 minutes, and then washed three times with PBS supplemented with 0.2% BSA. Labeled *B. burgdorferi* were resuspended in BSK-H medium (Sigma-Aldrich, St. Louis, MO) containing 20% glycerol, with a final spirochete density of 1-2 × 10^8^ per ml, and stored in aliquots at −80°C.

### Attachment of radiolabeled *B. burgdorferi* to mammalian cells

Binding of *B. burgdorferi* to mammalian cells was quantified according to procedures described previously [62]. One or two days prior to the assay, mammalian cells were lifted and plated in 96-well break-apart microtiter plates coated with 2 μg/ml *Yersinia pseudotuberculosis* recombinant purified invasin protein [113]. On the day of the experiment, frozen aliquots of radiolabeled *B. burgdorferi* were thawed and resuspended in 1.8 ml of BSK-H medium without serum and then incubated for 2 hours at room temperature to allow for physiologic recovery of the bacteria. *B. burgdorferi* were then diluted 1:3 in 10 mM HEPES, 10 mM glucose, 50 mM NaCl (pH 7.0). The spirochetes were checked for intact morphology and vigorous motility. Before adding bacteria, the confluent monolayer of mammalian cells was washed twice with PBS. To promote host cell-bacterium contact, the microtiter plates were centrifuged at 190 × g for 5 minutes at 23-24°C and then gently rocked at 24°C for 1 hour. Unbound bacteria were removed by washing the monolayers three times in PBS supplemented with 0.2% BSA. Cells integrity was checked microscopically and bound bacteria were quantified by scintillation counter. Four replicates were used for each treatment in these experiments.

To determine the effect of enzymatic removal of GAGs from host cells surface on *B. burgdorferi* attachment, the monolayers were incubated at 37°C for 2 hours with 0.5 U/ml of heparinase I (H2519), or chondroitinase ABC (C3667) (Sigma-Aldrich, St. Louis, MO) in RPMI 1640 supplemented with 1% BSA, 10^-2^ trypsin inhibitory units per ml of aprotinin, and 150 μg/ml of phenylmethylsulfonyl fluoride (PMSF). The monolayers were washed twice with PBS, and then binding assay with the radiolabeled bacteria was conducted as described above. All binding experiments were conducted at least three times and data from one representative experiment are presented in the Figures 1 and 2. T-test for samples with unequal variance was used to determine if inhibition of binding of *B. burgdorferi* after a specific treatment was statistically significant relative to the Mock treatment.

### PCR-amplification of major known plasmid-borne genes encoding virulence factors of *B. burgdorferi*

The genes encoding virulence factors that have been identified by several researchers previously were amplified by PCR using *Taq* DNA polymerase under the following conditions: initial denaturation at 95°C for 2 minutes, 35 cycles of denaturation at 94°C for 1 minute, annealing at 40°C or 50°C for 1 minute, extension at 65°C for 1 minute, and final extension at 72°C for 10 minutes. Genomic DNA of B31 and N40D10/E9 strains were used as PCR templates. Primers were designed based upon published B31 sequences [101] and are listed in Additional file 1: Table S1.

### Southern hybridization of genomic DNA of B31 and N40D10/E9 strains digested with *Eco*RI with *bbk32* gene as a probe

Genomic DNA of B31 and N40D10/E9 strains were digested with *Eco*RI enzyme overnight at 37°C and digested DNA was resolved by agarose gel electrophoresis. DNA in the gel was then transferred to a Nytran SPC nylon membrane (Whatman, Piscataway, NJ) in alkali transfer buffer (0.4 M NaOH). The *bbk32* gene was amplified from the B31 strain by PCR as described above. The resulting PCR amplicon was labeled with digoxigenin-dUTP by random priming. DIG high prime DNA labeling and detection starter kit II (Roche Applied Science, Indianapolis, IN) was used for probe preparation, Southern hybridization, and immunological chemiluminescent signal detection. All procedures were conducted according to manufacturer’s instruction.

### Two-dimensional electrophoresis of total proteins from B31 and N40D10/E9 strains

Fifty micrograms of total proteins extracted from *B. burgdorferi* strains B31 and N40D10/E9 were lyophilized and redissolved to 1 mg/ml in 1:1 diluted SDS boiling buffer:urea sample buffer before loading. Two-dimensional electrophoresis was performed using the carrier ampholine method of isoelectric focusing [114,115] by Kendrick Labs, Inc. (Madison, WI). Isoelectric focusing was carried out in a glass tube of inner diameter 2.3 mm using 2% pH 4–8 mix Servalytes (Serva, Heidelberg Germany) for 9,600 volt-hrs. Fifty nanograms of an IEF internal standard, tropomyosin was added to the sample. This protein migrates as a doublet with lower polypeptide spot of MW 33,000 and pI 5.2.

After equilibration for 10 min in Buffer 'O' (10% glycerol, 50 mM dithiothreitol, 2.3% SDS and 0.0625 M tris, pH 6.8), each tube gel was sealed to the top of a stacking gel that overlaid a 10% acrylamide slab gel (0.75 mm thick). SDS slab gel electrophoresis was carried out for about 4 hrs at 15 mA/gel. The following proteins (Sigma-Aldrich, St. Louis, MO) were used as molecular weight standards: myosin (220,000), phosphorylase A (94,000), catalase (60,000), actin (43,000), carbonic anhydrase (29,000) and lysozyme (14,000). These standards appear along the basic edge of the silver-stained [116] 10% acrylamide slab gel. The silver stained gels were dried between sheets of cellophane with the acid edge to the left side.

Duplicate gels were obtained from each sample and were scanned with a laser densitometer (Model PDSI, Molecular Dynamics Inc, Sunnyvale, CA). The scanner was checked for linearity prior to scanning with a calibrated Neutral Density Filter Set (MellesGriot, Irvine, CA). The images were analyzed using Progenesis Same Spots software (version 4.0, 2010, Nonlinear Dynamics) and Progenesis PG240 software (version 2006, Nonlinear Dynamics, Durham, NC). Selected spots were cut out and limited MALDI mass spectrometric (MALDI-MS) analyses were conducted at the Protein Core Facility of Columbia University at New York.

### In-gel digestion of proteins

Gel spots were transferred to clean tubes, water was added to completely hydrate gels, and the plastic coating was removed with clean tweezers. Gel spots were prepared for digestion by washing twice with 100 μl of 0.05 M Tris, pH 8.5/30% acetonitrile for 20 minutes with shaking, then with 100% acetonitrile for 1–2 min. After removing the washes, the gel pieces were dried for 30 minutes in a Speed-Vac concentrator. Gels were digested by adding 0.08 μg modified trypsin (sequencing grade, Roche Molecular Biochemicals) in 13-15 μl 0.025 M Tris, pH 8.5. The tubes were placed in a heating block at 32°C and left overnight. Peptides were extracted with 2X 50 μl of 50% acetonitrile/2% TFA; the combined extracts were dried and resuspended in matrix solution.

### MALDI-MS analysis

Matrix solution was prepared by making a 10 mg/mL solution of 4-hydroxy-α-cyanocinnamic acid in 50% acetonitrile/ 0.1% TFA and adding two internal standards, angiotensin and ACTH 7–38 peptide, to the matrix solution. The dried digest was dissolved in 3 μl matrix/standard solution and 0.5 μl was spotted onto the sample plate. When the spot was completely dried, it was washed twice with water. MALDI-MS analysis was performed on the digest using an Applied Biosystems Voyager DE Pro mass spectrometer in the linear mode.

### Peptide mass search

Average peptide masses were entered into search programs to search the NCBI and/or GenPept databases for a protein match. Programs used were Mascot at http://www.matrixscience.com and MS-Fit at http://prospector.ucsf.edu. Cysteine residues were modified by acrylamide.

Parameters for web-based search using Mascot were as follows: Database: NCBI; Taxonomy: bacteria; Variable modifications: Oxidation (M), Carboxyamidomethyl (C); Missed cleavages: 2; Error tolerance for Peptide average masses: 0.5 Da. Parameters for web-based search using MS-FIT were as follows: Database: NCBI; Taxonomy: bacteria; Constant mods: Possible mods: Oxidation of M; Minimum number of peptides to match: 4.

### Mouse model of infection

Four-week old C3H/HeN female mice (Charles River Laboratories, Wilmington, MA) were inoculated subcutaneously on the top of the right hind leg on the dorsal side at a dose of 10, 10^2^, 10^3^ or 10^4^*B. burgdorferi* strain B31 or N40D10/E9 in each mouse with the first two dose groups containing three mice each. Higher doses of infection (10^3^ and 10^4^ per mouse) were used to inoculate two mice each. After 14 days of infection, mice were euthanized and blood collected. Skin at the inoculation site, ear as a site for disseminated skin infection, heart, urinary bladder, and one joint were transferred to tubes containing BSK-II medium supplemented with 6% rabbit serum and antibiotic mixture for *Borrelia* (Sigma-Aldrich, St Louis, MO) and grown at 33°C. The median infectious doses (ID_50_) for B31 and N40D10/E9 were determined by examination of cultures from the mouse tissues.

Joint disease severity was determined by measuring the diameters of the tibiotarsal joints with a caliper and pictures taken. For histological examination, joints of infected mice were fixed in neutral buffered formalin, processed by routine histological methods, and scored blindly for arthritis severity, as described [117]. This work was conducted by the histology core facility of New Jersey Medical School. UMDNJ-New Jersey Medical School is accredited (Accreditation number 000534) by the International Association for Assessment and Accreditation of Laboratory Animals Care (AAALAC International), and the animal protocol used was approved by the Institutional Animal Care and Use Committee (IACUC) at UMDNJ.

## Abbreviations

GAGs: Glycosaminoglycans; PFGE: Pulsed Field Gel Electrophoresis; RST: rRNA spacer types; Osp: Outer surface protein; HI: HeparinaseI; ECM: Extracellular Matrix; Chon. ABC: Chonndroitinase ABC; MVLST: multi-virulence locus sequence typing.

## Competing interests

Authors of this manuscript have no competing financial or personal interests or relatioships with any organization.

## Authors’ contributions

NP and KC designed the research; KC and MA conducted the experiments; NP, KC and SWB analyzed and interpreted data; and KC and NP wrote the paper. All authors read and approved the manuscript.

## Supplementary Material

Additional file 1 ** Primers used for PCR amplification of the specific genes encoding virulence factors of *B. burgdorferi.*** Click here for file
