# Supplementary material for: Comparative molecular analyses of Borrelia burgdorferi sensu stricto strains B31 and N40D10/E9 and determination of their pathogenicity
Source: BMC Microbiol. 2012 Jul 30;12:157. doi: 10.1186/1471-2180-12-157 (PMC3511255; doi:10.1186/1471-2180-12-157)
Supplement: Additional file 1 — Primers used for PCR amplification of the specific genes encoding virulence factors of B. burgdorferi. [file 1471-2180-12-157-S1.pdf]

Additional Table 1. Primers used for PCR amplification of the specific genes encoding virulence factors of *B. burgdorferi.*

Gene	Sequence	Tm(^{0}C)
bb0153	ATGTTTAAGCTGCCAGAACTT	59.5
	CTAATTAATCACTTCATTGTAAACTTTTG	60.2
bb0184 (*csrA*)	ATGCTAGTATTGTCAAGAAAAGCA	60.7
	TTAATTTTCATTCTTGAAATAATGATTA	59.0
bb0219	ATGATAAAATCAATTTTAGATTATT	53.2
	TTAACCTAGTGAAACATCGAG	56.0
bb0268	ATGTTTATAAGCAGGGAATTGA	59.0
	TTATTCCTTCTTGCTCATTAAAG	58.2
bb0383(*bmpA*)	ATGAATAAAATATTGTTGTTGA	53.0
	TTAAATAAATTCTTTAAGAAACTTC	53.3
bb0647 (bosR)	ATGAACGACAACATAATAGACGT	59.0
	TCATAAAGTGATTTCCTTGTTC	57.3
bba25 (*dbpB*)	TGTAGTATTGGATTAGT	40.4
	ACTAATCCAATACTACA	40.4
bba68 (*cspA*)	TTGAAAAAAGCCAAACTAAATAT	57.6
	TTAGTAAAAGGCAGGTTTTAAAG	57.4
bbd14	ATGATAATAAAAATAAAAAATAATGTC	54.2
	AATTTTGATTAATTTTAATTTTGCTGATTCTTTTAAAACCT	69.1
bbd18	ATGCAAAAAGAAATAACAATAAACTAT	58.3
	TTAAATTTTGGTTTTTTCCCC	60.4
bbe22 (*pncA*)	TATAAATAGTAGTTTTCTAATTAAATACTATTGAA	56.9
	AATAATTTCTTTGATTAACCAACTTCAAAATTA	64.2
bbg02	GTGGAAATAAATTTACAAAGTAAAT	55.5
	CTATTTTTTTTGTATGCCAATT	56.5
bbh06 (*cspZ*)	ATCTCTAAAGATTTTAGCAGGGGAGAA	65.0
	CCTTGTGATCTATAATAAAGTTTGCTTAATAG	62.3
*bbj*09 (*ospD*)	ATGAAAAAAATTAATAAAAATACT	49.9
	TTAAGTATTTAACAAGGCCACA	57.9
*bbk*17 (*adeC*)	ATGGATTTATTTAAAATTGAAGCTA	58.0
	TTAATAATCTACAAAACAAAAAGAATC	57.0
bbk32	ATGATTTATTCATAAGATATGAA	51.5
	TTAGTACCAAACGCCATTCTTGTCAATGA	70.8
bbu06	AATTAAGTATGTGGAGGATATATATATGAGCA	62.6
	TCCTTTACTTTTAATTTAATTTCTTTCATTATCATATTTAG	65.1
vlsE1	TTTCAAGTGCAATTTTATTAACAACTTTCT	64.4
	GCAGCTTCAACAATCTCCTTTAT	62.3
*bb0147*flank	TACCGTTAAGCGCATGAAAGATCAAGAAA	71.6
	CCCTACTCAAAGCAAACTCCTCAATAAGCC	71.7
